# How did the use of the social marketing approach in Egyptian communities succeed in improving breastfeeding practices and infants’ growth?

**DOI:** 10.1186/s12889-024-18469-y

**Published:** 2024-05-13

**Authors:** Ammal M. Metwally, Walaa A. Basha, Ghada A. Elshaarawy, Sara F. Sallam, Inas R. El-Alameey, Amira S. El Rifay, Walaa Yousef, Amira A. Goda, Galal A. Elashry, Doaa E. Ahmed, Nayera E. Hassan, Sahar A. El-Masry, Nihad A. Ibrahim, Soha M. Abd El Dayem, Wafaa A. Kandeel, Ebtissam M. Salah El-Din, Rokia Abd Elshafy S. El Banna, Iman H. Kamel, Enas M. Abdelhamid, Mohamed Abdelrahman, Walaa S. Mahmoud

**Affiliations:** 1 Community Medicine Research Department, Medical Research and Clinical Studies Institute, National Research Centre (Affiliation ID: 60014618), Dokki, Cairo, Egypt; 2 Biological Anthropology Department, Medical Research and Clinical Studies Institute, National Research Centre (Affiliation ID: 60014618), Dokki, Cairo, Egypt; 3 Child Health Department, Medical Research and Clinical Studies Institute, National Research Centre (Affiliation ID: 60014618), Dokki, Cairo, Egypt; 4 Clinical Nutrition Department,Faculty of Applied Medical Sciences, Taibahu University, El Medina, Saudi Arabia; 5 Department of Food Contaminants and Toxicology, Food Industry and Nutrition Research Institute, National Research Centre (Affiliation ID: 60014618), Dokki, Cairo, Egypt; 6 Pediatrics Departtment, Medical Research and Clinical Studies Institute, National Research Centre (Affiliation ID: 60014618), Dokki, Cairo, Egypt; 7 Faculty of Biotechnology, Medical Biotechnology Department, Misr University for Science and Technology MUST, Giza, Egypt; 8 Public Health and Community Medicine, Medical Research and Clinical Studies Institute, National Research Centre (ID: 60014618), Dokki, P.O. 12622, Giza, Egypt

**Keywords:** Social marketing (SM) approach, Early breastfeeding initiation, Exclusive breastfeeding, Breastfeeding continuation till 2 years, Responsiveness to cues of hunger and satiety, Physical growth of infants

## Abstract

**Introduction:**

Improving breastfeeding practices does not always link to interventions relying only on improving nutrition awareness and education but needs cultural and behavioral insights **. **

**Aim:**

This study aimed to evaluate the changes in core breastfeeding indicators as a result of the use of social marketing (SM) approach for improving breastfeeding practices of Egyptian women and the physical growth of infants aged 6 to 12 months. The core breastfeeding indicators were: Early initiation of breastfeeding within one hour of birth, Predominant and exclusive breastfeeding to 6 months (EBF), Bottle feeding with formula, continued breastfeeding to 1 and 2 years, and responsiveness to cues of hunger and satiety.

**Methods:**

A quasi-experimental longitudinal study with a posttest-only control design was done over 3 years in three phases; the first was in-depth interviews and formative research followed by health education and counseling interventions and ended by measuring the outcome. Motivating mothers’ voluntary behaviors toward breastfeeding promotion “feeding your baby like a baby” was done using SM principles: product, price, place, and promotion. The interventions targeted 646 pregnant women in their last trimester and delivered mothers and 1454 women in their childbearing period. The statistical analysis was done by using SPSS program, version 26.

**Results:**

Most mothers showed significantly increased awareness about the benefits of breastfeeding and became interested in breastfeeding their children outside the house using the breastfeeding cover (Gawn) (*p* < 0.05). Breastfeeding initiation, exclusive breastfeeding under 6 months, frequency of breastfeeding per day, and percentage of children who continued breastfeeding till 2 years, were significantly increased (from 30%, 23%, 56%, and 32% to 62%, 47.3%, 69%, and 43.5% respectively). The girls who recorded underweight results over boys during the first year of life were significantly improved (*p* < 0.01) after the intervention (from 52.1% to 18.8% respectively). At the same time, girls found to be obese before the intervention (15.6%) became no longer obese.

**Conclusions:**

Improvement for the majority of the key breastfeeding indicators and physical growth of infants indicates that raising a healthy generation should start by promoting breastfeeding practices that are respectable to societal norms.

## Background

The World Health Organization (WHO) recommends that breastfeeding has to be initiated within the first hour after birth. Infants have to be exclusively breastfed for the first 6 months, with continued breastfeeding until 2 years of age or older [1] due to its well-known benefits for health, socioemotional and cognitive development [2–7].

Breastfeeding is directly linked to two of the United Nations Sustainable Development Goals (SDGs), SDG 2 and SDG 3, which focus on improved nutrition, maternal and child health. Indirectly improving breastfeeding practices will progress towards SDGs 4, 5, and 6 (breastfeeding impacts on intelligence, enhancing economic and human capital development), as well as SDG 10 by reducing inequality between the rich and poor [2, 4]. Egypt Vision 2030 is in line with SDGs as Egypt according to the Sixth pillar which is health is committed to progress towards ensuring protection for the vulnerable; children, women, and elderly [8]. Therefore, there is consensus agreement that exclusive breastfeeding is a priority area, with an increase of up to at least 50% of children being exclusively breastfed till 6 months by 2030 and to ensure universal coverage of this practice [9].

In the Middle East, the number of mothers who particularly breastfeed for six months is recorded to be 20.5% [10–12]. Meanwhile, the Egypt Demographic and Health Survey (EDHS) showed that the rate of exclusive breastfeeding dropped from 70 and 30% in 2008 [13] to less than 50% and 13% in 2014 [14] for infants under three months of age and 4–5 months of age respectively. Breastfeeding has been shown to significantly lower the risk of infections in childhood and adulthood and lower the risk of type 2 diabetes mellitus and obesity in adulthood [15]. EBF is essential for enhancing newborn nutrition on a worldwide scale, which will enhance health outcomes for all populations. There is data evidence that sustained breastfeeding is associated with a reduced risk of mortality during the neonatal period all over the world irrespective of the studied countries [16–18]. In Egypt, the current infant mortality rate in 2024 is 12.816 deaths per 1000 live births, with a 2.66% decline from 2023 [19]. Unfortunately, no recent available data regarding infant mortality rate in rural areas with either old published data [20] or being restricted to one government [21]. The infant mortality rate was higher in rural as opposed to urban areas according to data published in 2010 [22]. However, the association between breastfeeding and the under-five mortality (U5M) was documented [23]. The U5M risk is 69% less among children who have ever breastfed compared with children who never breastfed. In Egypt, there are variations in U5M being higher in rural areas compared to urban ones.

In Egypt, the understanding of why many mothers experience difficulties in maintaining exclusive breastfeeding till six months of life is still a gap [24]. World Health Organization (WHO) recommended breastfeeding counseling for overcoming difficulties in optimal feeding practices [25–28]. Addressing any encountered barriers, required besides the educational and promotional programs, a supportive positive environment to make healthy options available. From this perspective, the current study proposed providing breastfeeding counseling through a different approach, that is the use of social marketing (SM). SM is defined as the usage of marketing to design and apply programs to promote socially beneficial behavior change [29] and to achieve consistent changes in population behaviors [30]. Accordingly, the current study focused on improving breastfeeding practices among Egyptian women by counseling mothers through social marketing principles as a new strategy. The study evaluated the effectiveness and the success of using SM principles of promoting “feeding your baby like a baby” as an approach to improve breastfeeding practices and the physical growth of infants aged up to 2 years. This study targeted changes for eight core indicators as primary behavioral change outcomes [24] by the use of SM principles: Early initiation of breastfeeding within one hour of birth, exclusive breastfeeding (EBF) to 6 months, continued breastfeeding at 1 year, continued breastfeeding at 2 years, children ever breastfed, predominant breastfeeding under 6 months, and responsiveness to cues of hunger and satiety. While a feeding style that responds to infants’ cues of hunger and satiety promotes infants’ ability to naturally self-regulate their food intake later on in life, healthier infant weight could also be achieved if their mothers actively respond to these cues [31]. So, the possibility of the success of the use of SM principles for increasing responsiveness to cues of hunger and satiety has been also investigated as a challenging change to achieve.

## Methods

### Study design

The study was an interventional evaluation study with a before-and-after comparison conducted over three years’ duration starting from Jan. 2017 till Jan. 2020. A quasi-experimental design with posttest-only control design was done. Two villages were included in the study as intervention and control villages. This design was chosen to prevent possible contamination if the other village was chosen as a control before the intervention so that the real impact could be assessed.

### Study setting and participants

#### The intervention village

El Othmanyia village of El Mahallah El Kubra district– Gharbyia governorate was selected as the site of the project implementation. El Othmanyia village total married women in the childbearing period (19–45 years) were 2100. The control village was chosen as a matched group from the control village (Nemra el Basal village). The Economic Research Forum and CAPMS in Egypt have developed a wealth index that is based on the socio-economic status and was used for the classification of the socioeconomic status of the villages [32, 33]. Using the human development index, both villages were selected from the same governorate and the same district to have the same sociodemographic status, but in different local village unit (one in the east and the other in the west of El Mahallah El Kubra district) to have similar socioeconomic status.

The selected villages have powerful and active well-organized community-based associations with on-ground community health workers (CHWs) who were trained and serve as key players for the provision of motivational messages to the target groups.

### Target groups

**Fig. 1:**
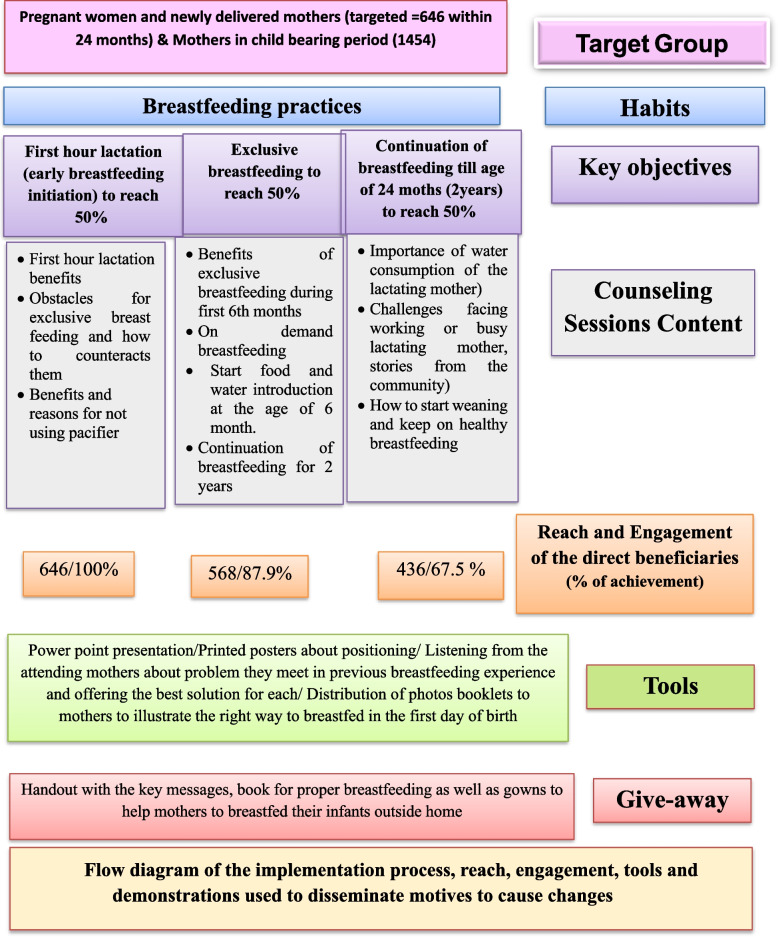
Flow diagram of the implementation process, reach, engagement, tools and demonstrations used to disseminate motives to cause changes

The direct beneficiaries for the intervention included pregnant women in their third trimester to help them have the best start and initiate their breastfeeding as soon as possible after birth, as well as mothers of newly born (at birth). Accordingly, a total of 646 mothers of infants were enrolled in the study along 2 years of follow-up. Women in their childbearing period (future mothers) were also targeted as indirect beneficiaries (*n* = 1454). A total number of 2100 women, including women who intended to breastfeed their infants and those who did not and who signed informed consent forms, were targeted for the intervention. The age range of the women enrolled in the study was 19–45 years. The total number of mothers who engaged in the study were those who completed the intervention sessions; completed the 10 messages within three months of the interventions and engaged in the quarterly follow-up visits within two years of intervention (Fig. 1). Engaged mothers were 436 out of 646 enrolled (67.5%).

### Phases of the study

The study was conducted in phases; the first was the formative assessment research followed by the interventions phase and ended with evaluation for measuring the outcome and impact.

#### Phase one; assessment phase

Participant in-depth interviews and formative research were used to collect data from the beneficiaries. The in-depth interview focused on listening to the attending mothers about problems they met in their previous breastfeeding experience and offering the best solution for each. Community consultation to help identify the “Best practices”, those appropriate, convenient, and suitable with the local culture and capabilities was also conducted. Meanwhile, formative research was used to assess the current situation of breastfeeding practices and identify the behaviors to be targeted, pricing, and promotional messages. The formative research also identified the factors that influenced mothers’ decisions to breastfeed and those that hindered or encouraged women to breastfeed. All practices were determined according to the designed pre-tested questionnaire.

#### Phase two; intervention phase

The interventions included capacity building of the activities’ implementers by the research team specialists from the National Research Center of Egypt which lasted for 4 months. The training was directed to 7 community health workers (CHWs); 5 nurses and 2 physicians of the rural health unit of the intervention village. So that they became community educators delivering the right messages about proper breastfeeding practices to all participants. They were targeted by the educational toolkit developed by the related researchers which was tailored more specifically to their needs and respected their norms according to the findings of the assessment phase.

Health education and counseling interventions targeted both the direct and the indirect beneficiaries. The longitudinal interventions followed over 646 mothers from early pregnancy throughout their infant’s second year of life.

Counseling sessions related to the importance, early initiation, and continuation of mothers to successfully breastfeed their infants and identifying the warning signs that need breastfeeding consultant intervention were delivered over 12 months to target the primary beneficiaries. For each mother 10 messages that are related to the targeted breastfeeding indicators for change [1] were delivered over 3 months; 3 messages were covered in the first session during the first month and 4 messages were added and covered in the second session during the second month and 3 different messages were added during the third session during the third month. Then, this is followed by repeating the same messages every 3 months. Accordingly, during 12 months of implementation, 4 rounds were conducted to cover delivering the messages to all targeted participants. Concerning the direct beneficiaries, the first set of messages was attended by 646 pregnant and delivered mothers. The second set of messages was attended by 568 mothers, and the third by 436 mothers. The activation process for the targeted primary and secondary groups with follow-up was conducted quarterly for 24 months for each mother. The flow diagram of the educational interventions’ implementation process, messages, reach, engagement, tools, giveaways, and demonstrations used to disseminate motives to cause change, is illustrated in Fig. 1.

Pregnant women and newly delivered mothers who were reached were 646 of which 436 were engaged in the counseling sessions and were covered by all messages for 24 months with an engagement rate of 67.5% (Fig. 1). The major reason for the dropout is the engagement of women in household and fieldwork activities.

The interventions were based on the use of a social marketing approach. This approach was based on using the motives for successful breastfeeding practices and overcoming the detected obstacles and factors contributing to declining of breastfeeding practices for improving breastfeeding practices over one year of interventions. A set of interventional messages concerning proper weaning practices was directed to our beneficiaries and its results were published [34].

#### Phase three; evaluation phase

Two methodological approaches were used during the evaluation; the first was by comparing the conducted practices as a result of the intervention between the intervention villages with those who did not receive any intervention in the non-intervention village. The second was through looking for direct evidence: Assessing the change in breastfeeding practices as well as the growth before and after the interventions within the intervention village which means that the study group served as self-control.

The end-of-study evaluation was done once the interventions had been completed through using a participatory approach by CHWs under the supervision of the study team. To measure the intervention's effectiveness, mothers and their infants were assessed and evaluated by the same structured questionnaires to test the breastfeeding practices and infants' growth before and after the interventions.

### Basis for sample size calculation during the assessment and evaluation phases

A sample size of 141 houses produced a two-sided 90% confidence interval with a width equal to 0.100 when the sample proportion for exclusive breastfeeding up to 6 months of age is 0.130 [14] which was rounded to 150 households [35, 36]. Houses were randomly selected out of the houses of the direct beneficiaries who engaged in the intervention (*n* = 436 mothers). Houses were selected by a systematic random sample. A total number of 200 women who were residents in these houses and who fulfilled the selection criteria were enrolled in the assessment and evaluation stages together with their infants aged 6 months up to 12 months. All infants of eligible mothers were included (one or 2 infants). For the rest of the mothers who were enrolled in the study (210 of 646 mothers) but did not engage in completing the intervention, their initial data were discarded, and they were excluded from being evaluated.

**Table:** Confidence interval formula: exact (Clopper Pearson)

**Confidence Level**	**Sample Size (N)**	**Target Width**	**Actual Width**	**Proportion (P)**	**Lower Limit**	**Upper Limit**	**Width if ** ***P*** ** = 0.5**
0.900	141	0.100	0.100	0.130	0.086	0.186	0.145

### Key Performance Indicators (KPIs):

The core KPI which are the study primary outcome to evaluate the success of the interventions were defined according to the World Health Organization, 2019 [37]:

1. Early initiation of breastfeeding
2. Exclusive breastfeeding (EBF) under 6 months
3. Continued breastfeeding at 1 year
4. Continued breastfeeding at 2 years
5. Children ever breastfed
6. Predominant breastfeeding under 6 months
7. Bottle feeding with formula
8. Percentages of responsiveness to cues of hunger and satiety

Exclusive breastfeeding: The infant has received only breast milk from his/her mother or a wet nurse, or expressed breast milk, and no other liquids or solids with the exception of drops or syrups consisting of vitamins, mineral supplements, or medicines [1].

Predominant breastfeeding “allows” Oral Rehydration Salts (ORS), vitamin and/or mineral supplements, ritual fluids, water and water-based drinks, and fruit juice. Other liquids, including non-human milk and food-based fluids, are not allowed, and no semi-solid or solid foods are allowed [1].

### Interventions targeted the control group within the control village were social by design

The well-trained community members who were the local people engaged in the implementation process of the intervention village, act as educators for the social workers of the control village. Such engagement was a social dynamic that fostered continuous engagement which worked towards sustainability and the continuation of the process resulting in lasting and continued positive behavior change. All participants from the control village received the educational toolkit developed for promoting breastfeeding practices as well as the giveaways (Fig. 1).

### Social marketing (SM) approach

Health education through using social marketing principles [29]: The implementation of the market plan focused on increasing the reach and engagement of mothers to have a profound impact and sustained success of breastfeeding. The marketing plan included the following:

#### Target behaviors (outcome)

1. Increase breastfeeding early initiation rates
2. Increase breastfeeding duration rates to 2 years
3. Increase the rate of EBF under 6 months
4. Increase general public support for breastfeeding practices

#### Product(s)

The Product is breastfeeding. The choice of breastfeeding benefits that has been emphasized was identified during formative research according to each targeted behavior.

The formative research indicated that the emotional benefits to the baby were emphasized more than the health benefits be more influential with mothers.

#### Price

For addressing the price factor, the marketing plan focused on interventions to lower the identified costs (barriers to breastfeeding and faulty beliefs) or make them more acceptable.

Encouragement of responsive feeding of mothers to their babies’ cues of hunger and satiety is free of charge. The following guiding principles during breastfeeding were provided and all are free of charge; feed directly or assisted, feed slowly and patiently, encourage feeding without forcing and avoid distractions while talking and looking at the child.

Moreover, behavioral change through the SM approach was applied to overcome the identified barriers by teaching the health providers to increase the awareness and concern of the mothers of breastfeeding's motives (benefits) and help mothers develop ways to lower the costs (barriers) most relevant to them personally.

#### Place

The Place factor was addressed by using the governmental public health facilities (the village rural health unit) and the village community-based associations to make these public settings more welcoming to breastfeeding women. Moreover, nursing women were also reached at their homes through the CHWs.

Interventions to facilitate support for breastfeeding practices through professional training directed towards nurses, physicians, and health workers at the rural health unit as well as the village community-based association’s members so that they could discuss breastfeeding with family members and friends.

#### Promotion and motives

The promotional messages targeted factors detected to motivate and deter from encouraging women to breastfeed. The promotion messages used were out of the reported motives by ever breastfed mothers (190 mothers) that were expressed by more than half of the mothers; mainly: Save money, time, and effort, increase the mother-baby bond, and boost the child's immunity. In addition, reasons for not using the pacifier were also added to the motives.

#### Slogan

The slogan of promotion to breastfeeding “feed me like a baby” was used for message distribution. The messages were converted to posters and cards. The content of the message although being derived from the women themselves and socially responsive was tested through 4 focus group discussions each with 10 women. The four selected focus groups were: Pregnant women, mothers of infants 0–6 months, mothers of infants 6–24 months, and women in the childbearing period.

The messages were from babies to their mothers “*One thing I wanted to tell you, my mom, once I'm out feed me like a baby. Breastfeed me for the 1st 6 months, and don't give anything else before I'm 6 months, continue breastfeeding me for up to two years. The only food I will love is the one coming from you. When you breastfeed, me you will not only save money, time and effort but also increase my immunity. I feel you and you feel me in your heart, please do not use the pacifier as it makes me vulnerable to infection. LOVE you, mom.*"

#### Change support process

Some tactic points were used to support the SM approach through:

- The village’s pediatricians were requested to encourage breastfeeding over other substitutes while providing recommendations for the mothers
- Nurses were trained to help mothers on how to start and overcome the initial challenges of breastfeeding.
- Distributing maternity messages on breastfeeding tips for moms who have Android mobiles using the WhatsApp application.
- Mothers were introduced to the competition, those who joined the competition received free breastfeeding covers (gown) to encourage them to breastfeed in public.

### Methods

A well-structured questionnaire was applied to the target groups through an interview to collect the nutritional data. The Centers for Disease Control (CDC) Infant Feeding Practices Questionnaire [38] was used to measure feeding practices throughout the first year of life. The main topics covered in the questionnaire included: Food fed to the infant, including breast milk and infant formula, patterns of breastfeeding, solid food intake, and other complementary foods and liquids were recorded. In addition, the timing for initiation of breastfeeding, the duration for exclusive breastfeeding or predominant breastfeeding or bottle feeding with formula, and the responsiveness to cues of hunger and satiety were also assessed and evaluated. Moreover, factors that may contribute to infant feeding practices and breastfeeding success and other issues (e.g. experiences with breast pumps and pacifiers) were also investigated.

Growth assessment was done for each infant aged 6–12 months twice (before and after the interventions) using anthropometric parameters.

All anthropometric measurements were taken by using standard anthropometric protocols and standardized instruments. Z scores (WAZ, HAZ, and WHZ) were calculated for each infant. They were measured, following the recommendations of the “International Biological Program” [39]. Infant Body Mass Index (BMI) was calculated: weight (in kilograms) divided by height (length) (in meters squared). The infant’s BMI percentile was calculated for age and sex based on the Egyptian Growth Reference Charts [40]. The standard deviation score (Z score); to exclude the effect of age; was calculated for the weight (WAZ), height (HAZ), and BMI (BMI-Z) according to the Egyptian Growth Curve by the following equation [41].

$$\mathrm{Z \,score}=\frac{{{\text{individual}}}^{\mathrm{^{\prime}}}\mathrm{s \,variable}-\mathrm{ mean \,value \,of \,reference \,population \,SD \,of \,the \,reference \,population}}{\mathrm{SD \,of \,the \,reference \,population}}$$

The researchers, who are anthropologists, took anthropometric measurements on the infants, including weight, supine length, occipitofrontal circumference (head circumference HC), and mid-upper arm circumference (MUAC) on the left arm. A flexible, non-stretchable measuring tape was applied to assess measurements to the nearest 0.1 cm, following standardized research protocols and Quality control measures elaborated for the World Health Organization Multicenter Growth Reference Report [42]. Each infant was also examined by the Holtain Body Composition Analyzer (Computerized Holtain Body Composition Analyzer -Holtain’s BCA, Holtain Ltd., UK, Wales, Crosswell No.646512). The following parameters were derived: the percentage of body fat (Fat %: an estimate of the proportion of fat to the total body weight), fat mass (FM: an estimate of the fraction of the total body weight that is adipose tissue), fat free mass (FFM: an estimate of the fraction of the total body weight that is not adipose tissue) [43] and Total Body Water (TBW: an estimate of the fluid occupies intracellular and extracellular spaces, comprising about 0.6 L/kg (63.3%) of body mass) for hydration assessment [44, 45].

### Data quality assurance

Data quality was controlled through condensed training sessions about how to conduct the questionnaire in a standardized way given to the data collectors. Data collection was conducted under the supervision of professional team members from the National Research Centre of Egypt (NRC) and their appropriate supervision that was carried out by the National Research Centre team members. A pilot study was performed on 10% of the questionnaire to ensure the validity of the questionnaire items by revising and modifying difficulty-understood items or language and then re-introducing them. The results of the pretest were not included in the study.

### Setting targets for the interventions

According to the assessment results, the target objectives were set to be realistic and achievable as follows: For any of the studied breastfeeding (BF) indicators, if its measure was 25% or less, the target was set to reach 50%. For any indicator whose measure was in the range of 25- 50%, the target was set to reach 75%. For any indicator whose measure was > 50% and < 75%, the target was set to reach 70–80%. For any indicator whose measure was > 75%, the target was at least to sustain the indicator.

### Statistical methods

After data cleaning, all completed questionnaires were entered into the computer. Statistical analysis was done by using the Statistical Package of Social Software program (SPSS), version 26. Data were presented as quantitative and qualitative variables. The quantitative variables included: the mother's age, the number of children in the house, body circumferences, and body composition parameters. The qualitative variables included: the mother's education, occupation, marital status, awareness of the meaning of exclusive breastfeeding and its benefits, awareness of initiating breastfeeding during the first hour of delivery, awareness of the meaning of colostrum milk and its benefits, intention of start weaning, intention of continued breastfeeding, consumption of liquids in the first day of delivery and among children < 6 m, consumption of solid, semi-solid or soft food among children < 6 m, breastfeeding and bottle feeding, breastfeeding during illness, breastfeeding initiation during the first hour of delivery, exclusive breastfeeding under 6 months, continued breastfeeding, frequency of breastfeeding per day, children ever breastfed, WAZ, WLZ. For quantitative variables, the data were summarized using descriptive statistics, where mean and standard deviation were used. However, numbers and percentages were used for qualitative values. Before and after the intervention, related indices were compared for the studied group. The following tests of significance were used: Pearson’s Chi-square test (χ2) and Z test (for qualitative data), paired t-test (for continuous data between the pre and post-interventions), and t-test (for continuous data between means of two groups). Percent change or mean change was measured for the differences between pre and post-intervention, where + ve good indicator or -ve bad indicator means improvement in these indicators and vice versa. A *P*-value less than 0.05 was considered statistically significant, while a *P*-value less than 0.01 was considered highly statistically significant.

## Results

A total of 200 mothers of the targeted mothers who completed the whole intervention process of one year and followed up on their BF practices for another year were included.

*Sociodemographic characteristics* of the direct beneficiaries between the intervention and the control villages are shown in Table 1. The differences between the characteristics of the participants within the two villages were not statistically significant suggesting that the participants within the two villages were balanced and comparable with each other. The age range of the mothers in the intervention village (19–30 years) was slightly insignificantly higher than that of the control village (20–30 years). The majority of mothers had middle school education; preparatory or secondary education (62.0% in the intervention village and 65.5% in the control village). The majority were married housewives and had an average of three children under 12 years old.

**Table 1:** Sociodemographic characteristics of the participants between the intervention and the control villages

Sociodemographic data	Intervention village (*n* = 200)	Control village (*n* = 200)	*P* value
	**n (%)**	**n(%)**	
**Mother's age (years)**			
*Range*	19—30	20—30	
*Mean* ± *SD*	23.5 ± 6.3	24.5 ± 5.1	0.096
**Mother Education ** *n (%)*			
Illiterate/	21 (10.5%)	17(8.5.0%)	
Read and Write/ Primary	28 (14.0%)	32(16.0%)	
Preparatory	55 (27.5%)	71 (35.5%)	
Secondary	69 (34.5%)	60 (30.0%)	0.356
University	27 (13.5%)	20(10.0%)	
**Mother Occupation ** *n (%)*			
Housewife	188 (94%)	177 (88.5%)	
Farmer	6 (3%)	10 (5%)	
Worker	2 (1%)	6 (3%)	0.246
Professional	4 (2%)	7(3.5%)	
**Marital status of mothers ** *n (%)*			
Married	194 (97%)	186 (93%)	
Widow	5 (2.5%)	9 (4.5%)	0.136
Divorced	1 (.5%)	5 (2.5%)	
**Number of children in the house (< 12 years)**			
*Range*	1—5	1—5	0.12
*Mean* ± *SD*	2.8 ± 2	3.1 ± 1	

t test between means, X^2^ between groups

### Breastfeeding indicators

A comparison of breastfeeding awareness, attitude, and practices indicators between the intervention and control villages among the direct beneficiaries, who are the mothers of infants from birth to 2 years, was shown in Table 2. The results revealed that for all the studied indicators concerning awareness, attitude, and practices, the intervention group performed significantly better than those in the control group and before the intervention. Table 2 showed also the effect of the interventions on both the predominant breastfeeding under 6 months and those who are EBF; the formal one decreased significantly to half from 46.0% to 18%. The proportion of children who were EBF raised from 23.0% to 47% with a 24.0% improvement versus a 28% reduction of the predominant breastfeeding as a result of the interventions. Continue breastfeeding during illness was also significantly increased from 77.9% to 87.9%. The initial assessment of the studied women revealed many faulty practices as the consumption of liquids, solids, semi-solid, or soft food among children less than 6 months (46%) and using the pacifier among children less than 2 years (72.0%). These faulty practices were significantly corrected as a result of the interventions.

**Table 2:** Frequency comparison of breastfeeding awareness, attitude and practices indicators between intervention and control villages among the participants before and after intervention

Indicators^c^	Intervention village		Percent change	Control village *N* = 200 n(%)	*P* value of Z test Pre vs Post Intervention	*P* value of Z test Post Intervention vs Control
	**Pre- intervention ** ***n*** ** = 200** **n(%)**	**Post- intervention ** ***n*** ** = 200** **n (%)**				
	**Awareness Indicators**					
**Aware of the meaning of exclusive breastfeeding**	96(48.0)	148(74.0)	26.0%	87(43.5)	** < 0.001** ^**^	** < 0.001** ^**^
**Aware of the benefits of breastfeeding:**						
Know 2 benefits	156(78.0)	90(45.0)	-33.0%	164(82.0)	** < 0.001** ^**^	** < 0.001** ^**^
Know ≥ 3 benefits	44(22.0)	110(55.0)	33.0%	36(18.0)	** < 0.001** ^**^	** < 0.001** ^**^
***P*** ** value of X** ^**2**^	** < 0.001** ^a**^			** < 0.001** ^b**^		
**Aware of initiating breastfeeding during the first hour of delivery**	106(53.0)	164(82.0)	29.0%	110(55.0)	** < 0.001** ^**^	** < 0.001** ^**^
**Aware of the meaning of colostrum milk**	166(83.0)	186(93.0)	10.0%	157(78.5)	** < 0.001** ^**^	** < 0.001** ^**^
**Aware of the benefits of colostrum milk:**						
Know 2 benefits	170(85.0)	84(42.0)	-43.0%	175(87.5)	** < 0.001** ^**^	** < 0.001** ^**^
Know ≥ 3 benefits	30(15.0)	116(58.0)	43.0%	25(12.5)	** < 0.001** ^**^	** < 0.001** ^**^
***P*** ** value of X** ^**2**^	** < 0.001** ^a**^			** < 0.001** ^b**^		
	**Attitude Indicators**					
**Intention of start weaning at age:**						
≤ 4 months	76(38.0)	14(7.0)	-31.0%	82(41.0)	** < 0.001** ^**^	** < 0.001** ^**^
5- 6 months	124(62.0)	186(93.0)	31.0%	118(59.0)	** < 0.001** ^**^	** < 0.001** ^**^
***P*** ** value of X** ^**2**^	** < 0.001** ^a**^			** < 0.001** ^b**^		
**Intention of continued breastfeeding:**						
One year	94(47.0)	60(30.0)	-17.0%	114(57.0)	**0.001** ^**^	** < 0.001** ^**^
One and half years	40(20.0)	50(25.0)	5.0%	34(17.0)	0.230	0.050
2 years	66(33.0)	90(45.0)	12.0%	52(26.0)	0.014*	** < 0.001** ^**^
***P*** ** value of X** ^**2**^	**0.002** ^a**^			** < 0.001** ^b**^		
	**Practices Indicators**					
**Consumption of liquids in the first day of delivery**	126(63.0)	73(36.5)	-26.5%	129(64.5)	** < 0.001** ^**^	** < 0.001** ^**^
**Consumption of liquids among children < 6m** ^**d**^	69(46.0)	27(18.0)	-28.0%	56(37.3)	** < 0.001** ^**^	** < 0.001** ^**^
**Consumption of solid, semi-solid or soft food among children < 6m** ^**d**^ **:**						
≤ 4 months	33(22.0)	5(3.3)	-18.7%	30(20.0)	** < 0.001** ^**^	** < 0.001** ^**^
5- < 6 months	36(24.0)	22(14.7)	-9.3%	26(17.3)	**0.040** ^*^	0.529
***P*** ** value of X** ^**2**^	**0.008** ^a**^			**0.002** ^b**^		
**Breastfeeding and bottle feeding:**						
Exclusive Breastfeeding	46(23.0)	94(47.0)	24.0%	40(20.0)	** < 0.001** ^**^	** < 0.001** ^**^
Predominant Breastfeeding (Breastfeeding & Fluids)	92(46.0)	36(18.0)	-28.0%	74(37.0)	** < 0.001** ^**^	** < 0.001** ^**^
Bottle feeding	16(8.0)	0(0.0)	-8.0%	6(3.0)	** < 0.001** ^**^	** < 0.001** ^**^
Mixed (Breastfeeding and Bottle feeding)	46(23.0)	70(35.0)	12.0%	80(40.0)	** < 0.004** ^**^	0.153
***P*** ** value of X** ^**2**^	** < 0.001** ^a**^			** < 0.001** ^b**^		
**Breastfeeding during illness** ^**e**^ **:**						
Continue breastfeeding	148(77.9)	174(87.9)	10.0%	147(75.8)	**0.008** ^**^	**0.002** ^**^
Stop breastfeeding and bottle feeding or special food	42(22.1)	24(12.1)	-10.0%	47(24.2)	**0.008** ^**^	**0.002** ^**^
***P*** ** value of X** ^**2**^	**0.013** ^a*^			**0.002** ^b**^		
**Using pacifier**	144(72.0)	57(28.5)		134(67.0)	** < 0.001** ^**^	** < 0.001** ^**^

^*^significant < 0.05

^**^highly significant < 0.01

^**a**^ *P* value of X^2^ between before and after interventions

^**b**^ *P* value of X^2^ between after interventions and control

^c^Percentages were shown in parentheses

^**d**^Out of children aged from 6 m- < 2y: in village before interventions (*n* = 150), in village after interventions (*n* = 150), and in control village (*n* = 150)

^**e**^ Out of mothers ever breastfed: in village before interventions (*n* = 190), in village after interventions (*n* = 198), and in control

As shown in Table 3, all targeted objectives as a result of the interventions were achieved regarding breastfeeding initiation during the first hour of delivery, EBF under 6 months, and frequency of BF per day which doubled their values at the baseline, except for continued breastfeeding till 2 years which showed some lag behind achievement. Moreover, 56.0% of children from 0–2 years received breastfeeding when they got hungry, or on-demand and this percentage significantly raised to 69.0% as a result of the interventions with a significant difference from that of the control village for all indicators. A strong improvement was observed for the breastfeeding initiation during the first hour of delivery as a result of the intervention (% change of 32).

**Table 3:** Target versus achievements of breastfeeding practices’ indicators among the participants in the intervention village before and after the intervention

Indicators^c^	Intervention village			Percent change	Control village *n* = 200 n(%)	*P* value of Z test Pre vs Post Intervention	*P* value of Z test Post Intervention vs Control
	**Pre- intervention ** ***n*** ** = 200** **n(%)**	**Target Objectives as a result of the interventions**	**Post- intervention ** ***n*** ** = 200** **n (%)**				
**Breastfeeding initiation during the first hour of delivery**	60(30.0)	**required to be doubled** to reach 60–70%	124(62.0)	32.0%	92(46.0)	**0.001** ^**^	**0.001** ^**^
**Exclusive Breastfeeding under 6 months** ^**d**^	46(23.0)	**required to be doubled** to reach 46–50%	71(47.3)	24.3%	30(20.0)	**0.001** ^**^	**0.001** ^**^
**Continued breastfeeding till:**		**Continued breastfeeding till 2**	65(32.5)	-19.0%	123(61. 5)	** < 0.001** ^**^	** < 0.001** ^**^
One year	103(51.5)	**years required to be motivated** to reach 50%	48(24.0)	7.5%	27(13.5)	0.061	**0.007** ^**^
One and half years	33(16.5)		87(43.5)	11.5%	50(25.0)	**0.018** ^*^	** < 0.001** ^**^
2 years	64(32.0)						
***P*** ** value of X** ^**2**^	**0.001** ^a**^				**0.001** ^b**^		
**Frequency of Breastfeeding per Day** (breastfeeding when they get hungry or on demand)	112(56.0)	**required to be motivated** to reach 65%—75%	138(69.0)	13.0%	100(50.0)	**0.003** ^**^	**0.001** ^**^
**Children Ever Breastfed**	190(95.0)	**Support sustainability**	198(99.0)	4.0%	194(97.0)	**0.009** ^**^	0.077

^*^significant < 0.05

^**^highly significant < 0.01

^**a**^ *P* value between before and after interventions

^**b**^ *P* value between after interventions and control

^**c**^percentages were shown in parentheses

^**d**^ Out of children aged from 6 m- < 2y: in village before interventions (*n* = 150), in village after interventions (*n* = 150), and in control village (*n* = 150)

### Growth indices

*A c*omparison of the growth indices among the children of direct beneficiaries in the intervention village pre and post-intervention were shown in Table 4. 180 infants aged 6–12 months (84 boys and 96 girls) whose mothers engaged and completed the interventions for one year were assessed and evaluated. Improvement was observed significantly for the majority of the growth parameters as a result of the interventions. Growth indices including weight-for-age Z score (<—2 SD) as an indicator for underweight, weight-for-height Z score (<—2 SD) as an indicator for wasting; and weight-for-age Z score (> + 2 SD) as an indicator for overweight/ obesity, are presented in Table 4. It was obvious that before the intervention phase, girls recorded to be underweight were higher than boys. However, strong significant improvements as a result of interventions were observed mainly for girls who were underweight before the intervention with a reduction of 33.3% versus 4.8% reduction among boys. It was obvious that after intervention wasting disappeared completely in both boys and girls. The same results were recorded for obesity but for only girls with a highly significant difference ((*P* < 0.001). Concerning other growth parameters, a highly significant difference was noticed regarding MUAC. A significant decrease in both FM and fat % and a significant increase in FFM and TBW were shown as a result of the intervention. Meanwhile, both the HC and MUAC were within the normal ranges before and after the interventions.

**Table 4:** Comparison of growth indices for the infants aged 6–12 months in the intervention village pre and post the intervention

Growth indices^a^	Pre- intervention (*n* = 180)	Post- intervention (*n* = 180)	Percent change or mean change	*P* value
**WAZ (Underweight)**				
Boys (84) *n (%)*	10 (11.9)	6 (7.1)	-4.8%	0.294
Girls (96) *n (%)*	50 (52.1)	18 (18.8)	-33.3%	** < 0.001** ^**^
Total (180) *n (%)*	60 (33.3)	24 (13.3)	-20.0%	** < 0.001** ^**^
**WLZ (Wasting)**				
Boys (84) *n (%)*	3 (3.6)	0 (0.0)	-3.6%	0.080
Girls (96) *n (%)*	2 (2.1)	0 (0.0)	-2.1%	0.156
Total (180) *n (%)*	5 (2.8)	0 (0.0)	-2.8%	0.024*
**WLZ (Overweight)**				
Boys (84) *n (%)*	18 (21.4)	30 (35.7)	14.3%	0.063
Girls (96) *n (%)*	23 (24.0)	18 (18.8)	-5.2%	0.379
Total (180) *n (%)*	41 (22.8)	49 (26.7)	3.9%	0.332
**WLZ (Obese)**				
Boys (84) *n (%)*	10 (11.9)	6 (7.1)	-4.8%	0.294
Girls (96) *n (%)*	15 (15.6)	0 (0.0)	-15.6%	** < 0.001** ^**^
Total (180) *n (%)*	25 (13.9)	6 (3.3)	-10.6%	** < 0.001** ^**^
**Body circumferences**				
HC (cm) *Mean* ± *SD* ^*b*^	46.91 ± 3.0	48.68 ± 2.76	1.77 ± 0.24	0.133
MUAC (cm) *Mean* ± *SD* ^*c*^	15.10 ± 2.90	18.77 ± 2.22	3.67 ± 0.68	** < 0.001** ^**^
**Body composition parameters** [46]				
TBW(Kg) *Mean* ± *SD*	7.90 ± 4.40	12.92 ± 4.30	5.02 ± 0.1	0.001**
FFM (Kg) *Mean* ± *SD*	8.61 ± 3.97	11.09 ± 1.80	2.48 ± 2.17	0.010*
FM (Kg) *Mean* ± *SD*	6.0 ± 7.0	3.10 ± 1.11	-2.90 ± 5.89	0.027*
Fat% *Mean* ± *SD*	31.03 ± 19.4	21.75 ± 5.96	-9.55 ± 13.44	0.003**

*WAZ* Weight-for-age Z score (<—2 SD) as an indicator for underweight. *WLZ* Weight-for- Length Z score (<—2 SD) as an indicator for wasting; (> + 2 SD) as an indicator for overweight/ obese, *FM* Fat mass, *FFM* Fat free mass, *TBW* Total body water

^*^significant < 0.05

^**^highly significant < 0.01

^a^Percentages were shown in parentheses out of row

^b^Normal range of HC ( -2 SD TO 2 SD) = 37.6- 47.2, Median = 47.05

^c^Normal range of MUAC ( -2 SD TO 2 SD) = 12.4- 15.7, Median = 13.8

## Discussion

Exclusive breastfeeding (EBF) is a public health priority globally. According to the World Health Organization (WHO) [1], the global prevalence of EBF during the first six months of life remains low particularly in low- and middle-income countries [47]. In Egypt, breastfeeding practices are not always optimal. The prevalence and determinants of EBF during the first six months of life were done among 16 Northern African countries. The estimated pooled prevalence of EBF did not reach the WHO target of 50% for the prevalence of EBF in the first six months which is listed among the six global nutrition targets by 2025.

The body lays the foundation for the future growth and development of a child during the first 2 years of life. Any nutritional deficiencies during this time can be manifested in the form of impaired cognitive development, and physical growth [48, 49] compromised educational achievement, and ultimately low economic productivity later in life [50]. Furthermore, the role of breastfeeding in boosting child immunity was evident and the weaknesses of eating habits decreased child immunity and were considered as an entry point to COVID-19 [51] and as a trigger for increasing phobia [52]. Investing in children's growth and development could theoretically drive the transformation endeavored for by 2030 under SDG3, i.e., good fitness and well-being for all [53–55].

The current study was conducted in phases; formative research was conducted to understand the motives and barriers for breastfeeding and detect obstacles and factors contributing to the decline of breastfeeding practices. The intervention phase employed a social marketing approach to reduce physical barriers to exclusive breastfeeding, to be continued for infants up to two years of age, and to facilitate for the mothers to breastfeed in public and making it easier, more convenient, and appropriate for all women. Initiating breastfeeding early within one hour of birth, and encouraging responsive feeding of mothers to their babies’ cues of hunger and satiety were also among the goals of this phase.

Breastfeeding judgments are influenced by a variety of variables, such as experience, perception, behaviors, and motivation [34, 53], When compared to pre-intervention and the control rural area, knowledge of the nature of exclusive breastfeeding and that colostrum is the first breast milk that includes antibodies to protect the infant from diseases increased dramatically after the intervention. Increased understanding of behaviors related to breastfeeding practices was found to be an assuring factor for enhancing breastfeeding according to recent Eastern Mediterranean studies [53–56] declaring that both early breastfeeding initiation and exclusive breastfeeding are common practices in some Arab countries even without promotion interventions. Their figures are comparable to the present study findings which showed significant improvement after the interventions (from 30% to 62.0% for the early breastfeeding initiation and from 23% to 47.3% for EBF less than 6 months). The World Health Organization considers early initiation of breastfeeding rates ranging from 50 to 89% and exclusive breastfeeding rates above 60% as good and as global targets set to be met by 2030 [57]. In Egypt, recent studies revealed similar results and that early breastfeeding was successfully initiated in the 1st hour in 29% of neonates, 55% and 82% in the first 24 h [58, 59].

Cultural factors were also reported by many studies in Middle Eastern countries; in which EBF’s primary barriers were women’s perceptions of insufficient breast milk, beliefs about infant need for water [60], or due to receiving conflicting advice such as encouraging herbal drinks [61]. Our study reported the barriers behind not giving colostrum to babies after birth to be due to some cultural beliefs. One of these beliefs is that “women who delivered by cesarean section (CS), her breast milk got spoiled due to the anesthesia and must not breastfeed except after 2 or 3 days”. Another belief is that “giving liquid to the babies less than 6 months is essential to relieve baby colic and constipation”. The reported cultural barriers against EBF were “breastfeeding should be done only inside the home (no breastfeeding outdoors)”, and “Mothers should not breastfeed their children during the night to save the milk for the day and for not to cause any stomach troubles for their babies”. The improvement of these two indicators occurred after the intervention pointed out the success of the SM approach in overcoming such barriers.

Responsive feeding of mothers to their babies’ cues of hunger and satiety raised from 31.2% to 68.8% as a result of this study’s interventions. This figure was slightly lower than that reported by two other Egyptian studies [62, 63]. The currently reported low percentage of responsive feeding of mothers to their babies’ cues of hunger and satiety before the interventions were attributed to the same reasons for not breastfeeding their infants exclusively under 6 months of age.

Due to the evidence of the successful role of community-based behavioral change interventions in many health promotion programs in Egypt, it is advisable to use the same approach for EBF promotion. The community-based behavioral change interventions succeeded in overcoming the cultural and local barriers; for endemic diseases [43–48], maternal health promotion [49, 50] and, child health promotion [51].

Continuation of breastfeeding till 24 months was sustained by less than half of the participants (45%). Meanwhile, this finding was more than that reported in the United Arab Emirates (U.A.E.) by a recent study in which only 28.7% of mothers were planning to continue breastfeeding for the child ≥ 24 months [56].

The strong social support that was applied through using the SM approach motivated not only the caregivers but also their mothers, mothers-in-law and their relatives. This support was one of the major reasons behind the great improvement achieved for the majority of the indicators. Moreover, faulty practices such as using the pacifier among infants less than 2 years, consumption of liquids, solid, semi-solid, or soft food among infants less than 6 months, and stopping breastfeeding during illness were high initially (72%, 46%, and 22.1% respectively). However, as a result of interventions, all these defective practices were significantly corrected and their percentages lowered to 28.5%, 18% and 12.1% respectively. The use of a pacifier was found to be a predictor of early termination of EBF at discharge [64–66].

Anthropometry has become a practical tool for evaluating the nutritional status of populations, particularly of children in developing countries [67]. Z-scores have been used in the present study to assess growth in children being the best method for analysis and presentation of anthropometric data [68]. Feeding infants with different milk formulas and the early introduction of solid foods were important factors in early life stages that favor the development of obesity in children [69]. Generally, child undernutrition is a global development problem resulting from poor access to nutritious foods, repeated infections, and inadequate maternal and child feeding and care practices during the first 1,000 days, from conception to age two [70]. In the first 1000 days of life, it was observed by some studies that boys reported having a higher prevalence of both wasting and stunting [71, 72].

In the present study, before interventions, girls recorded underweight results over boys which disappeared and significantly improved as a result of the interventions. A national study in Egypt on the determinants of stunting reported the importance of breastfeeding to have normal-weight children at their school age [51]. Wasting is an acute malnutrition condition and manifests when there is an absolute shortage of food [73]. The present study recognized well the critical importance of addressing wasting in both boys and girls as a result of the intervention. Childhood wasting requires urgent attention from policymakers and program implementers to reduce and maintain its percentage among young children at a level of less than 5% to achieve the sixth goal of Global Nutrition Targets 2025 for improvements of their nutritional status [74].

A better understanding of infant body composition patterns in addition to linear growth may present possible intervention opportunities to reduce the obesity risk in infancy and childhood, and possibly offset the risk of non-communicable disease in adulthood [75]. The first 6 months of life are a critical window for adiposity programming, as the risk for adiposity and cardiovascular diseases at age 21 years and beyond is associated with the rapid increase of weight detected in early life [76]. The current study focused on these body composition measures. The current study revealed a significant decrease in FM and fat % as a result of the intervention, proving the considerable benefits of breastfeeding to infants. This finding was consistent with a recent study, which found a decrease in fat mass index at 6 months in boys and girls who received exclusive breastfeeding at 3 months [77].

### Strengths of the study

The success of the use of the SM approach in promoting BF practices is the strength of our study as it respects societal norms. This study is of interest in the digital and social media influence era due to the following mentioned strengths. Our social marketing plan responded to local needs and clarified how to act on the behavior pathway by providing motives that worked as enablers contributing to the targeted behavior change goal to be then translated into changes in the daily and regular practice of mothers. Catchy, innovative messages were developed based on the detected motives that were in line with the village's local context and according to their cultural and behavioral insights. Countries with a similar context of limited resources can benefit from this model in which the concept and lessons learned from this study have to be used as a foundation for adaptation and replication.

### Limitations of the study

There were some limitations as a result of the used study design which is the quasi-experimental study. Since the baseline data for the comparison group (control group) was not collected before the implementation of the intervention for the comparison district, there are threats to the internal validity. Although our study methodology tried to ensure accurate outcomes with minimum measurement and social desirability bias, some sort of bias could be elicited on the outcome indicators. Another limitation was the difficulty in accessing infants 6–12 months in the control village resulted in limiting the measurements of the growth indices for the infants within the control village. Moreover, the elicited improvement in the physical indices could not be considered because of the improvement of the breastfeeding practices only but might be also because of the proper weaning practices that were advocated for during the intervention village.

## Conclusions of the study

To sum up, this study described a useful and promising social marketing approach able to improve healthy habits with the current results supporting the success of the use of social marketing principles for BF promotion. This program must be continued on a wider scale in different communities with similar contexts. The study findings reflect the importance of the use of the SM approach during counseling and education on BF practices in local communities. It is important to highlight that using the SM approach successfully corrected faulty practices such as the consumption of liquids, solids, semi-solids, or soft food among children less than 6 months and the use of pacifiers among children less than 2 years. Special interest should be directed to girls early in life even before being married, to give them advice about good and healthy patterns of feeding lifestyle.

## Data Availability

The datasets used and/or analyzed during the current study are available from the corresponding author on reasonable request.

## Abbreviations

BF: Breastfeeding
CDC: Centers for Disease Control
CHWs: Community health workers
CS: Cesarean section
EBF: Exclusive breastfeeding
EDHS: Egypt Demographic and Health Survey
FFM: Fat free mass
FM: Fat mass
HC: Head circumference
KPIs: Key Core performance indicators
MUAC: Mid-upper arm circumference
SDGs: United Nations Sustainable Development Goals
SM: Social marketing
SPSS: Statistical Package of Social Software program
TBW: Total Body Water
WHO: World Health Organization
